# Psychometric Properties of the Turkish Version of the Partners in Health Scale: Chronic Disease Self‐Management in Primary Healthcare

**DOI:** 10.1111/ijn.70007

**Published:** 2025-03-20

**Authors:** Julide Gulizar Yildirim, Sharon Lawn

**Affiliations:** ^1^ Faculty of Health Sciences, Department of Public Health Nursing Katip Celebi University Izmir Turkey; ^2^ College of Medicine & Public Health Department Flinders University Adelaide Australia

**Keywords:** chronic disease, confirmatory factor analysis, reliability, self‐management, validity

## Abstract

**Background:**

This study aimed to assess the psychometric properties of the Turkish version of the Partners in Health Scale (PIH‐TR), which was developed to assess the perceptions of patients with chronic conditions in primary care. Accurate assessment of facilitators and barriers to self‐management of chronic conditions, from the patients' perspective, is important for working effectively with them to promote better health outcomes.

**Methods:**

A cross‐sectional validation study was conducted and designed according to the STROBE guidelines. One hundred thirty‐six patients, aged 30–90 years (86.7% aged > 60), were recruited from family care centres. Data were collected using the revised PIH, an adapted version of Model‐2 (PIH‐TR), which is a 12‐item self‐rated measure of self‐management of chronic conditions. The PIH was translated into Turkish using Beaton et al.'s method. Content, construct validity and internal consistency analyses were undertaken to evaluate the data.

**Results:**

The PIH‐TR had satisfactory reliability and validity and revealed a four‐factor structure appropriate to the original scale: knowledge, partnership in treatment, recognition and management of symptoms and coping. Omega coefficient (0.860), test–retest reliability (0.841) and comparative fit indices (CFI) (0.99) were high.

**Conclusion:**

The PIH‐TR has good specifics and is reliable and valid as an objective self‐rated tool to assess self‐management of Turkish patients' chronic conditions.

**Summary**

What is already known about this topic?

- The Partners in Health (PIH) Scale supports collaboration between patient and clinician for self‐management care planning and is designed to facilitate the assessment of self‐management knowledge and behaviours of patients with chronic conditions.
- The PIH Scale facilitates individualied patient care and orients care focused on patient self‐management.
- Self‐management interventions enhance the patient's coping strategies in chronic disease management.

What this paper adds?

- The Turkish version of the PIH Scale was found to be a valid and reliable tool.
- The four‐factor structure and 12 items of the PIH‐TR have good internal consistency.

The implications of this paper:

- Patient and clinician engagement may have an important role in improving patient self‐management of chronic diseases.
- The PIH Scale can be used to evaluate self‐management interventions.
- The PIH‐TR measures four distinct factors related to self‐management of chronic conditions.

## 1 Introduction

Chronic diseases (CDs) constitute a major burden on healthcare systems, increase medical expenditure globally and contribute considerably to earlier than expected morbidity and mortality. The prevalence and incidence of lifestyle risk factors, which include physical inactivity, unhealthy diet and tobacco use, contribute to high rates of CDs, which account for seven of the 10 leading causes of death. CDs are responsible for 71% of all deaths globally, which represents an estimated 41 million deaths per year (WHO 2020).

Today, the increasing prevalence of CDs demonstrates the importance of CD control and community‐based disease management programmes (Battersby et al. 2015; Haslbeck et al. 2015; O'Connell et al. 2018; Riegel et al. 2021; Wilson et al. 2021). Chronic disease self‐management programmes (CDSMP) have been shown to have long‐lasting effects on patient self‐efficacy (Battersby et al. 2015). Self‐management interventions include patients working in collaboration with health professionals to develop attitudes, health behaviours and skills to cope with complex health conditions (Battersby et al. 2017). Integrating collaborative approaches provide an opportunity to coordinate the active involvement of patients in their own care and minimize the impact on CDs at the individual and community level (Battersby et al. 2017; Cheng et al. 2021; Wilson et al. 2021). The methods used to measure self‐management in the treatment of CDs are limited.

The Flinders Chronic Condition Management Program is a patient‐centred, collaborative patient–clinician self‐management care planning approach that provides individualized care, designed to facilitate the assessment of self‐management knowledge and behaviours of patients with chronic conditions, and individualize care from the patient's perspective, based on that assessment. The 12‐item Partners in Health (PIH) Scale was developed by a research group in Australia (Petkov et al. 2010; Smith et al. 2017) where this approach to CD management is used widely by its primary and secondary healthcare system. This scale was chosen for translation because there is no such measurement tool used in chronic condition self‐management in primary healthcare (PHC) in Turkey, and because it has few items, can be completed quickly and can be used in both primary and secondary care in Turkey. The study of validity and reliability of the PIH Scale was first conducted by Battersby et al. (2003), and analysis of a revised version of the tool (Model‐1) was conducted by Petkov et al. (2010). Lastly, following further revisions of the tool, Smith et al. (2017) tested the validity and reliability of the revised PIH (Model‐2), using Bayesian confirmatory factor analysis (CFA).

The PIH Scale has been used with several different population groups and within various contexts and translated into several languages, including Dutch, (Lenferink et al. 2016; Veldman et al. 2017), Spanish, (de Peñarrieta‐ Córdova et al. 2014), Chinese (Chiu et al. 2017; Xiaofei et al. 2017) and French (Hudon et al. 2019). The PIH Scale has been adapted for use in chronic obstructive pulmonary disease (COPD) (Lenferink et al. 2016), multiple chronic conditions (Battersby et al. 2015), Aboriginal populations with diabetes (Battersby et al. 2008), chronic renal disease (Baxter et al. 2017; Walker et al. 2014), older adults with hearing loss (Convery et al. 2018), severe mental disorders (Fotu and Tafa 2009) and liver cirrhosis (Ramachandran et al. 2021). To date, no Turkish language version of the PIH Scale is available.

### 1.1 Research Question

- Which PIH model (1 or 2) best matches the PIH‐TR and a Turkish‐speaking population sample?
- Do the newly adapted set of PIH‐TR (Turkish version) Scale items attain adequate reliability and validity (perform similar to the Australian revised version of PIH scale) (Model‐2)?

## 2 Methods

### 2.1 Study Aim

The aim was to evaluate the reliability and validity of the PIH Scale for Turkish populations by assessing the psychometric properties of the Turkish version of the Australian Partners in Health Scale (PIH‐TR).

### 2.2 Study Design

The cross‐sectional validation study examined the validity and reliability of the PIH Scale adapted for use with Turkish‐speaking populations. The reporting of findings is in accordance with the ‘STrengthening the Reporting of OBservational studies in Epidemiology’ STROBE checklist.

#### 2.2.1 Turkish‐Language Cross‐Cultural Adaptation

The translation and cultural adaptation of the PIH Scale followed established guidelines (Beaton et al. 2000; Hall et al. 2018). This involved six steps: forward translation, synthesis, backward translation, committee review and field testing of preliminary and final versions. Both the Model‐1 PIH Scale (Petkov et al. 2010) and Model‐2 revised PIH Scale (Smith et al. 2017) were included in this study.

For forward translation, two bilingual translators independently translated the PIH scale from English to Turkish. The translators' mother tongue was Turkish—one had a PhD (healthcare professional), she had worked as an English as a Second Language (ESL) tutor for more than 4 years at various language schools, and the other had a master's degree in English Language and Literature (ELL) Department. The translated (PIH‐TR) Model‐1 and Model‐2 versions were compared, and discrepancies, nuances and ambiguous meanings were highlighted. Subsequently, the PIH Scale was then translated backwards from Turkish to English by two sworn, registered translators independently (one was a native speaker of English and the other had a PhD in ELL).

After forward and backward translations were completed, the original and backward translations of both English and Turkish versions were then evaluated by an expert committee that comprised 10 teaching staff, each with PhD level qualifications—four in public health nursing, three in internal nursing, two in mental health nursing and one in health education and fieldwork. The experts reviewed all items and achieved equivalence of the original and target versions in four areas (semantic, idiomatic, experiential and conceptual). Finally, according to their suggestions, necessary changes were made to the scale items. For example, the Turkish equivalent of ‘deal with’ has the meaning of ‘cope with’. The Content Validity Index (CVI) was assessed using the Davis's technique (Polit 2014).

The preliminary scale was tested at two time points within a 2‐week time frame on a sample of 30 primary care Turkish patients aged 63.13 ± 11.64 (30–79 years) (Streiner et al. 2015). The sample included patients with diabetes, hypertension and arthritis. Test–retest reliability was evaluated by calculating the intra‐class correlation coefficient (ICC) values with a 95% confidence interval (CI), which for this study was 0.841, indicating high reliability (r = 0.859, *p* < 0.001) (Portney and Watkins 2015; Streiner et al. 2015). Lastly, a valid and reliable Turkish version of the PIH Scale, the PIH‐TR, was created for further testing with a larger sample. The details of methods used for this are provided below.

### 2.3 Sample and Sample Size

The study was conducted in the provincial city of the Izmir, Turkey. Patients who visited four PHC services between June 2019 and February 2021 were included using a convenience sampling method. This method which is a non‐probability sampling in which the researcher selects a sample based on ease of access to the patient population. PHC services were selected using a simple random sampling method in the Cigli and Karsiyaka regions. These regions were selected to represent both rural and urban areas. In scale development studies, the recommended number of participants' per‐item is 10 according to the Cochran formula (Polit 2014). The required sample size was therefore 120 participants, based on the recommendations of Polit (2014). The study sample was comprised of a total of 136 eligible CD patients, and the data were collected while the individuals were waiting for their appointment with a PHC provider.

Inclusion criteria were being a patient at the participating clinic, being at least 18 years old, being in good mental health, being a native Turkish speaker and being literate (able to read, write and understand Turkish language of at least a third grade level without diploma at primary school), having at least one CD (e.g., hypertension, diabetes, cardiovascular disease, osteoporosis, arthritis, COPD or asthma). Patients who were illiterate, whose mental health was currently unstable as assessed by their treating health professional or who were unable to communicate, were excluded from the study.

#### 2.3.1 Data Collection and Statistical Analysis

The author explained the research aim to participants, and the data were collected face‐to‐face by the researcher (JGY) who has field experience in the Family Health Care Center and a PhD degree in Public Health Nursing. The measure was completed in the allocated time of about 15 min.

Data were analysed using the Statistical Package for the Social Sciences 27.0 program (SPSS Inc., IBM, and Chicago, IL, USA). Descriptive statistics were performed on demographics using frequencies. The Kolmogorov–Smirnov test was applied to test normality by comparing the data to a normal distribution with the same sample mean and standard deviation of the sample (Polit 2014). Means, medians and standard deviations were calculated, and 95% CI were accepted. Results that showed a 95% CI (*p*‐value < 0.05) were considered statistically significant (Leech et al. 2015; Polit 2014).

The validation of the study's content (CVI), construct (CFA) and external validity (item means, item‐total correlation) were calculated. CVI and item‐total correlation was calculated taking into account the experts' views for content validity (Leech et al. 2015). Accordingly, ≤ 0.39 was categorised as weak; 0.40 ≤ α ≤ 0.69 considered as moderate; 0.70 ≤ α ≤ 0.89 as strong (Schober et al. 2018). The Pearson product–moment correlation coefficient was used for bivariate correlations between the scale and subscales.

For structural equation modelling of scale validity, CFA was carried out (Jöreskog et al. 2016; Leech et al. 2015; Polit 2014). The LISREL software program 8.8 (Scientific Software International, Inc., Lincolnwood, IL, USA) was used to complete the factor analysis of the PIH scale. CFA was used to examine the validity of the model in terms of both versions of PIH Scale (Model‐1) (Petkov et al. 2010) and the revised PIH Scale (Model‐2) (Smith et al. 2017) in order to evaluate the compatibility of the original 12‐item PIH Scale with the Turkish structure. The LISREL program includes goodness of fit indices: Chi‐square test, degrees of freedom (df), minimum fit function chi‐square, root mean square error of approximation (RMSEA ≤ 0.06) and 90% CI RMSEA, comparative fit indices (CFI), goodness of fit index (GFI), adjusted goodness of fit index (AGFI), normed fit index (NFI), Tuker–Lewis index (TLI ≥ 0.95), standardized root mean residual (S‐RMR ≤ 0.08) (Jöreskog et al. 2016; Kline 2016; Polit 2014; West et al. 2012).

The reliability analysis of the study (its internal consistency) was measured using McDonald's Omega and Cronbach's alpha for the PIH scale (Leech et al. 2015; McNeish 2018). When evaluating the reliability coefficient of a scale, experts suggest that the value of most items values should be above 0.70 to prove sufficiency (Polit 2014). Additionally, Spearman–Brown and the Guttman split‐half reliability coefficient applied for internal consistency. The Hotelling's *t*‐squared criterion test was used to test the suitability of the model (Pallant 2020). The ICC was calculated for test–retest reliability.

### 2.4 Measurements

The data were gathered using a socio‐demographic and chronic illness description form (12 questions) that was developed by the researchers informed by the literature and the PIH Scale (Appendix A). However, two different analysis methods (Models‐1 and ‐2) were applied in terms of item analyses. The 12‐item PIH Scale's four‐factor structure shows the patient's perception of each of the four following CDSMP domains: knowledge, partnership in treatment, recognition and management of symptoms and coping. Each scale item is scored using a 9‐point Likert scale (ranging from 0 to 8), with higher scores indicating *higher self‐management capabilities*. The total score ranges from 0 to 96, with 0 representing *poor self‐management* and 96 representing a *greater self‐management*. The response categories for items 1–4, 6, 8, 10–12 ranged from *very poor* (0 point) to *very good* (8 points) and for items 5, 7 and 9 from *never* (0 point) to *always* (8 points) (Petkov et al. 2010; Smith et al. 2017). For the PIH Scale version‐1 (Model‐1) questionnaire developed by Petkov et al. (2010), a total sum score and four subscale scores can be calculated: knowledge (items 1, 2, 4 and 8); adherence to treatment (items 3 and 5); symptom recognition and management (items 6, 7 and 9); coping (items 10–12). Cronbach's alpha was 0.82 for the total scale (Petkov et al. 2010). For the revised version of the PIH Scale (Model‐2) that uses a Bayesian CFA model, factor structure was changed as follows (Smith et al. 2017): knowledge (items 1 and 2); partnership in treatment (items 3–6), recognition and management of symptoms (items 7 and 8) and coping (items 9–12).

### 2.5 Ethical Considerations

In order to carry out the work, the research protocol was approved by the University Institutional Review Board for Non‐Interventional Clinical Studies Ethical Committee (IRB No: 144 date: 25.07.2017), and written permission was obtained from the institutions where recruitment to place. Verbal informed consent was obtained from the patients. This study complied with the Declaration of Helsinki.

## 3 Results

### 3.1 Participant Characteristics and Clinical Information

The socio‐demographic characteristics and CD status of the participants are shown in Table 1. The mean age of the patients was 69.61 ± 10.33 (30–90 years), with a CD duration of 12.38 ± 8.60 years (range, 1–45 years). The majority of patients were female (63.2%) and married (66.2%). Most of the patients (94.5%) reported having one type of CD (67.4%), 23% had two, and 9.6% had three types of CDs. Of those diagnosed with one type of CD, these included the following: Most had hypertension (36%), many had diabetes (13.1%), and some had arthritis (7.1%), cardiovascular disease (4.2%), thyroid (3.5%) or COPD/asthma (3.5%). Of those diagnosed with two types of CD, 14.3% had hypertension and diabetes, 2.9% had hypertension and asthma/COPD, 2.3% had hypertension or do and cardiovascular disease, and 3.5% had other types of disease (arthritis and thyroid). Of those who reported three types of CD, 5.1% of patients had hypertension, diabetes and cardiovascular disease, 2.9% had hypertension, diabetes and COPD, and 1.6% had hypertension, diabetes and arthritis. Patients reported that the median number of different type of medications they used was 1.0 ± 1.23 (range, 1–7 pieces). The general health perception of all the patients was moderate (2.48 ± 0.70) (range, 1–4 points).

**TABLE 1:** Demographic and clinical characteristics of participants (*N* = 136).

Descriptive characteristics	Number	Percentage
**Age (years)**		
30–59	18	13.2
60–70	52	38.2
71–90	66	48.5
**Gender**		
Female	86	63.2
Male	50	36.8
**Marital status**		
Single	46	33.8
Married	90	66.2
**Educational level**		
Literate	10	7.3
Primary school	60	44.1
Middle school	25	18.4
High school	31	22.8
University	9	6.6
**Social security**		
Private health insurance	6	4.4
National health insurance	125	91.9
None	5	3.7
**Socioeconomic status**		
Excess income	19	14.0
Budget balance	107	78.7
Expenses exceed income	10	7.4
**Employment status**		
Working	10	7.4
Retired	99	72.8
Unemployed	27	19.9
**Diagnosis of chronic illness**		
One type	91	66.9
Two types	31	22.8
Three types and over	14	10.3
**Duration of chronic illness (years)**		
Under 5 year	39	28.7
6–10	39	28.7
11–20	41	30.1
21–45	17	12.5
**Number of medications used (pcs)**		
1–3	118	86.8
4–7	17	12.5
Over 7	1	0.7
**Presence of social support in his/her family**		
Agree	105	77.2
Disagree	16	11.8
Living alone	15	11.0
**General health status**		
Worst	10	7.4
Middle	62	45.6
Good	56	41.2
Best	8	5.9

### 3.2 Validity Analysis Results

#### 3.2.1 Content Validity

Ten experts reviewed the PIH Scale to assess the content validity of linguistically approved version. The CVI for this study was 0.90 (r = 0.92). This result confirms the scale items give the meaning of the items in the original scale.

#### 3.2.2 Construct Validity

CFA was performed for construct validation; it allows more precise tests of an instrument's factor structure (Leech et al. 2015). Factors are the variables that to explicate the types of research question, can be reduced to a smaller number of factors using factor analysis. CFA enables the researcher to assign the items in an instrument to their respective factors according to theoretical expectations (Aroian and Norris 2005).

##### 3.2.2.1 CFA

**FIGURE 1:**
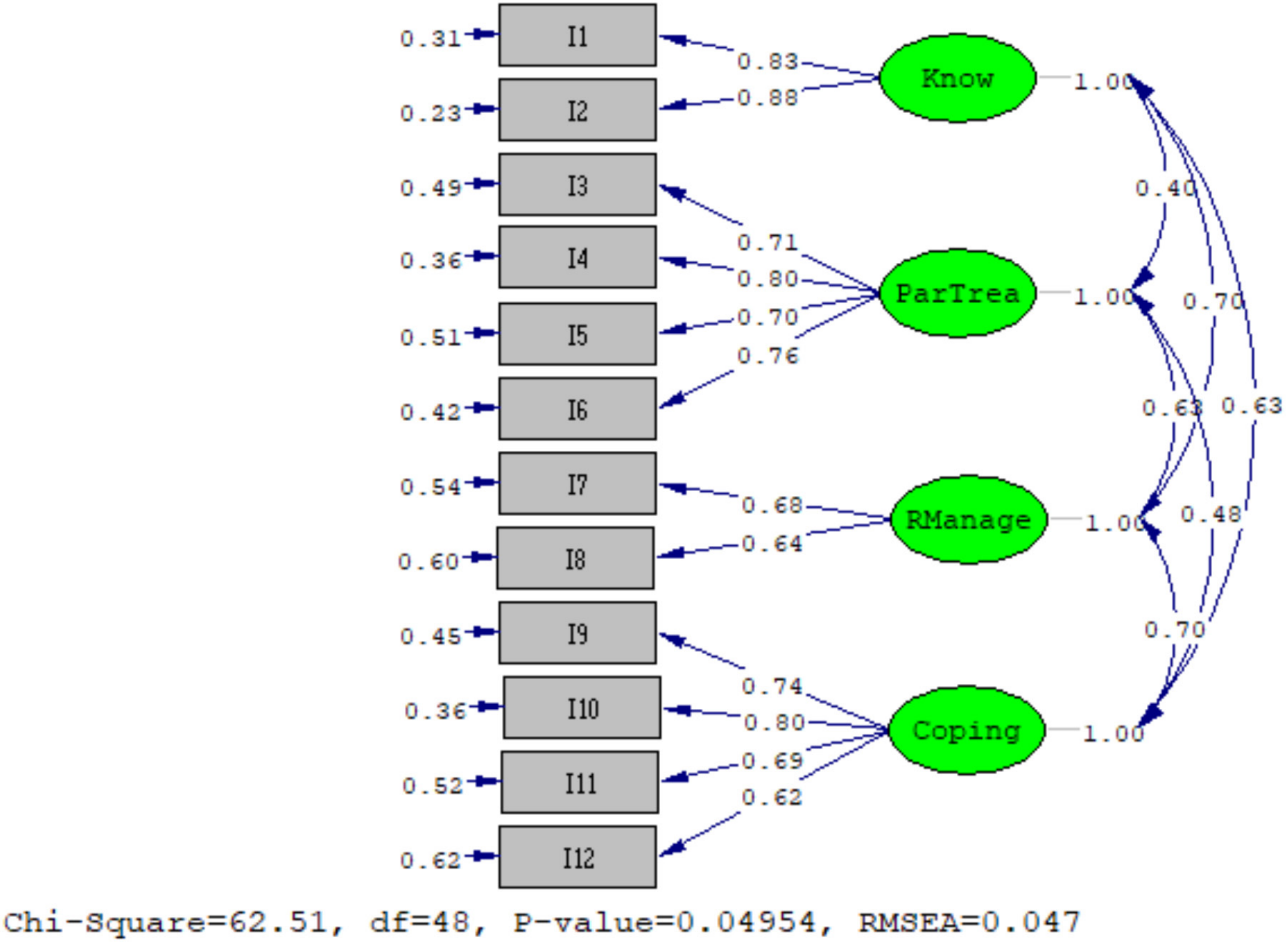
Structural equation model of Turkish Partners in Health Scale (standardized) (*N* = 136). I1–12 Item1–12; Know, Knowledge; ParTrea, Partnership in treatment; RManage, Recognition and management of symptoms.

Models‐1 and ‐2 goodness of fit indices are shown in Table 2. To begin, CFA was performed on the original PIH Scale (Model‐1) using the original factor structure. The goodness of fit indices for Model‐1 were generated (GFI: 0.78, AGFI: 0.65, CFI: 0.87, S‐RMR: 0.11, NFI: 0.84, TLI: 0.82), which indicated that the badness of fit indices. The Model‐1 fails the exact‐fit test at the 0.05 level (χ^2^(48) = 225.20, *p* < 0.001). RMSEA (0.165) showed that poor fit (90% CI [0.15, 0.19] *p* < 0.001) according to Kline (2016). Second, CFA on the revised PIH Scale (Model‐2) was generated with the following goodness of fit indices: GFI: 0.93, AGFI: 0.88, NFI: 0.95, TLI: 0.98, indicating that adequate the goodness of fit indices. RMSEA (0.047) showed adequate goodness of fit (90% CI [0.0, 0.074] *p* = 0.60) according to Kline (2016). The Model‐2 yielded a CFI of 0.99, S‐RMR of 0.049, and the Model‐2 passes the exact‐fit test at the 0.05 level (χ^2^(48) = 62.51, *p* = 0.049) (Table 2). Figure 1 shows the standardized solution values of a CFA model with 12 ordered categorical variables (I1–I12) measuring four factors. As a result, the hypothesis was accepted in the study, and the revised PIH Scale Model‐2 was accepted as valid for the Turkish version of the PIH Scale (PIH‐TR).

**TABLE 2:** Results of the confirmatory factor analysis models.

Index	Goodness fit criterion	Acceptable fit criterion	Finding of research for Original PIH Scale (Model‐1, 12 items, 4 factors)	Goodness or badness of fit index for Model‐1	Finding of research for revised PIH Scale (Model‐2, 12 item, 4 factor)	Goodness or badness of fit index for Model‐2
**RMSEA** (90% CI)	0.00 ≤ RMSEA ≤ 0.05	0.05 ≤ RMSEA ≤ 0.10	0.165 (0.15, 0.19)	Badness	0.047 (0.0, 0.074)	Goodness
**X** ^**2**^ **(df)**	—	—	225.20 (48)**	—	62.51 (48)*	—
**CFI**	0.95 ≤ CFI ≤ 1.00	0.90 ≤ CFI ≤ 0.95	0.87	Badness	0.99	Goodness
**NFI**	0.95 ≤ NFI ≤ 1.00	0.90 ≤ NFI ≤ 0.95	0.84	Badness	0.95	Goodness
**TLI**	0.95 ≤ TLI ≤ 1.00	0.90 ≤ TLI ≤ 0.95	0.82	Badness	0.98	Goodness
**S‐RMR**	0.00 ≤ SRMR ≤ 0.05	0.05 ≤ SRMR ≤ 0.08	0.11	Badness	0.049	Acceptable
**GFI**	0.95 ≤ GFI ≤ 1.00	0.90 ≤ GFI ≤ 0.95	0.78	Badness	0.93	Acceptable
**AGFI**	0.90 ≤ AGFI ≤ 1.00	0.85 ≤ AGFI ≤ 0.90	0.65	Badness	0.88	Acceptable

Abbreviations: *df*, degrees of freedom; *X* ^2^, Chi‐square; AGFI, adjusted goodness of fit index; CFI, comparative fit index; GFI, goodness of fit index; NFI, normed fit index, RMSEA, root mean square error of approximation; S‐RMR, standardized root mean square residual; TLI, Tucker–Lewis index.

** *p* < 0.001.

* *p* < 0.05.

##### 3.2.2.2 External Validity

For external validity analysis, item, scale means, standard deviations and mean of all inter‐item correlation coefficients are calculated. For reliability analysis, the PIH‐TR mean score was 26.12 ± 12.76 (1–66), the skewness value was 0.769 (Std. Error of skewness 0.208), and the kurtosis value was 0.924 (Std. Error of kurtosis 0.413). The PIH‐TR mean score showed a normal distribution. The lowest mean score for scale item 3 was 1.32 ± 1.36, and the highest for scale item 9 was 2.99 ± 1.87. The values of the total item correlations of the scale ranged from 0.428 (item 3) to 0.641 (item 10), and there was a high correlation between the lower factors, indicating that the features of the assessment tool influenced each other. The item‐total correlation coefficients were statistically significant (*p* < 0.001) (Table 3).

**TABLE 3:** Cronbach's alpha, estimates of McDonald's omega, *t*‐values and item‐total correlation for the PIH‐TR.

Factors	Items	Mean ± SD	*t*‐Values	*p*	Item‐total correlation	α if item deleted	α	ω if item deleted	ω
**Knowledge**	1. Overall, what I know about my health condition(s)	2.16 ± 1.46	4.21	< 0.001*	0.629	0.852	0.875	0.844	—
	2. Overall, what I know about the treatment, including medications of my health condition(s) is.	2.28 ± 1.44	2.99	< 0.001*	0.635	0.852		0.844	
**Partnership in treatment**	3. I take medications or carry out the treatments asked by my doctor or health worker	1.32 ± 1.36	6.64	< 0.001*	0.428	0.863		0.857	
	4. I share in decisions made about my health condition(s) with my doctor or health worker	1.52 ± 1.57	5.44	< 0.001*	0.603	0.853	0.802	0.847	0.807
	5. I am able to deal with health professionals to get the services I need that fit with my culture, values and beliefs	1.88 ± 1.46	6.77	< 0.001*	0.453	0.862		0.856	
	6. I attend appointments as asked by my doctor or health worker	1.55 ± 1.68	6.10	< 0.001*	0.484	0.861		0.857	
**Recognition and management of symptoms**	7. I keep track of my symptoms and early warning signs (e.g., blood sugar levels, peak flow, weight, shortness of breath, pain, sleep problems, mood)	2.74 ± 1.73	5.56	< 0.001*	0.537	0.857	0.623	0.849	—
	8. I take action when my early warning signs and symptoms get worse	2.54 ± 1.87	6.18	< 0.001*	0.511	0.859		0.851	
**Coping**	9. I manage the effect of my health condition(s) on my physical activity (i.e., walking, household tasks)	2.99 ± 1.87	6.23	<0.001*	0.579	0.854		0.846	
	10. I manage the effect of my health condition(s) on how I feel (i.e., my emotions and spiritual well‐being)	2.82 ± 1.84	5.40	< 0.001*	0.641	0.850	0.822	0.840	0.822
	11. I manage the effect of my health condition(s) on my social life (i.e., how I mix with other people)	2.32 ± 1.71	6.78	< 0.001*	0.530	0.858		0.850	
	12. Overall, I manage to live a healthy life (e.g., no smoking, moderate alcohol, healthy food, regular physical activity, manage stress)	1.99 ± 1.92	7.25	< 0.001*	0.592	0.854		0.844	
PIH‐TR mean score		26.12 ± 12.76	—	—	—	—	0.867	—	0.860

Abbreviations: α, Cronbach's alpha; SD, standard deviation; ω, McDonald's omega.

* *p* < 0.001.

### 3.3 Reliability Analysis

In this research, internal consistency reliability analyses used Cronbach's alpha, McDonald's Omega, Spearman–Brown, Guttmann split‐half reliability coefficient and Hotelling's *t*‐squared criterion. McDonald's Omega is a more comprehensive estimator of reliability, and it reduces to Cronbach's alpha under the assumption of essential tau‐equivalence (Hayes and Coutts 2020; McNeish 2018). Cronbach's alpha was 0., and the McDonald's Omega was 0.860 for all 12 items. Cronbach's alpha ranged between 0.623 and 0.875 for subscale items as shown in Table 3, and for the revised PIH Scale, Model‐2, Spearman–Brown correlation coefficient (0.755) and the Guttman split‐half coefficient (0.745) have high reliability, which indicate that this scale's items are homogeneous in terms of contents. Split‐half coefficient was 0.801 for the first half of the first six items, and 0.811 for the rest of the items, indicating high internal consistency. Hotelling's *t*‐squared criterion was found to be significant for the total PIH‐TR score (131.279 *p* < 0.001). This result showed that the patients did not perceive that individual items were asking the same thing (different ways of asking the same question) and that they responded to each PIH‐TR item directly reflecting on problems they experienced related to the item.

According to the PIH‐TR mean score and subscales scale information (see Table 4), the factor correlations of the PIH‐TR scores ranged between 0.366 and 0.837, which indicates that they are positively correlated through a weak to strong relationship. It was determined that the average of the total score of PIH‐TR was 26.12 ± 12.76. Another method showing the internal consistency of the scale is item analysis. In the study, item‐total score correlations of the scale were considered positively significant at a moderate level (from 0.428 to 0.641).

**TABLE 4:** PIH‐TR scale and total points of the subscale's correlations (*N* = 136).

	Min‐max	Average M ± SD	Knowledge	Partnership in treatment	Recognition and management of symptoms	Coping	Total PIH
**Knowledge (Know)**	0–15	4.44 ± 2.73	1				
**Partnership in treatment (ParTrea)**	0–23	6.27 ± 4.83	0.373**	1			
**Recognition and management of symptoms (RManage)**	0–14	5.28 ± 3.07	0.505**	0.432**	1		
**Coping**	0–32	10.13 ± 5.94	0.567**	0.366**	0.467**	1	
**Total PIH‐TR**	1–66	26.12 ± 12.76	0.740**	0.732**	0.729**	0.837**	1

Abbreviations: M, mean; SD, standard deviation.

** *p* < 0.001.

## 4 Discussion

In this study, the validity and reliability of the Turkish version of the PIH Scale, a validated self‐reported measure used to determine the self‐management, treatment needs and perceptions of individuals with CDs, were examined. This study explored whether the PIH‐TR Scale had good psychometric properties. Data were gathered demonstrating the scale's applicability to Turkish patients. This is the first study of the translation and adaptation of the Turkish version of this scale.

### 4.1 Validity

To examine the factorial structure of the scale, CFA was used for Model‐1 and Model‐2 (Aroian and Norris 2005; Brown 2015; Leech et al. 2015; Pallant 2020; Streiner et al. 2015). Initially, the precedence of Model‐1 results showed that the first model fit indices were unsatisfactory (NFI 0.84 TLI, 0.82). The analysis of the revised version of the PIH Scale, Model‐2, which was developed in accordance with the study by Smith et al. (2017), shows that the Turkish version of the scale is a valid measurement tool with good fit indices (Aroian and Norris 2005; Brown 2015; Jöreskog et al. 2016; Leech et al. 2015; Pallant 2020) and that the items included in the scale adequately represent the determined sub‐dimensions (NFI 0.95, TLI 0.98). Model‐2 was accepted as a valid and reliable Turkish PIH Scale. CFA supported the hypothesis that the PIH‐TR consisted of four factors: ‘knowledge’, ‘partnership in treatment’, ‘recognition and management of symptoms’ and ‘coping’. Petkov et al. (2010) found NFI value as 0.92, and CFI 0.95. For this study, the CFI value (0.99) and S‐RMR (0.049) are based on being a great fit. According to Kline (2016), the exact‐fit test indicates that the PIH‐TR, Model‐2 has better fit than other accepted translations of the PIH (χ^2^(48) = 62.51, *p* < 0.05). In contrast to the results of this study, the study adapting the PIH to Chinese language (Xiaofei et al. 2017) did not achieve goodness of fit (χ2(46) = 84.09, *p* < 0.001). The S‐RMR value (0.046) was desirable, CFI (0.965), and TLI (0.950) was a great fit.

In order to evaluate the self‐management of patients with CDs, the mean of the Turkish form of the scale was found to be 26.12 points. It was found to be different (78.1) from the mean found by the researchers in the adaptation process of the Dutch form of the scale (Lenferink et al. 2016), even lower than the mean score (82.17 points) in population sample for the French language translation of the PIH (Hudon et al. 2019). This result suggests that Turkish patients have lower self‐management abilities than Dutch‐ and French‐speaking patients. The presence of such different mean values in the Turkish study can be explained by cultural differences as well as differences in the participants' level of education, diagnosis of CDs, coping skills of individuals, the status of individuals who received training in disease management after diagnosis, engagement to CD management and duration of diagnosis.

### 4.2 Reliability

Since the PIH‐TR Scale is a Likert‐type scale, Cronbach's alpha coefficient, McDonald's Omega, Sperman–Brown, Guttman split‐half and Hotelling's *t*‐squared criterion test were used for the analysis of internal consistency (Leech et al. 2015; McNeish 2018; Polit 2014). The higher the coefficient of the scale, the more confident we are that the items in this scale are consistent with each other and consist with items that predict the items of the same characteristic (Polit 2014). The McDonald's omega was evaluated for this study and evaluated for the subscales of the PIH English version, which ranged between 0.70 and 0.95 for the subscales (Smith et al. 2017); however, in previous studies, Cronbach's alpha coefficient was evaluated in initial English version, French, Spanish, Dutch and Chinese versions (Hudon et al. 2019; Lenferink et al. 2016; de Peñarrieta‐ Córdova et al. 2014; Petkov et al. 2010; Xiaofei et al. 2017). The PIH‐TR had a good omega (0.860) and alpha coefficient (0.867), which showed that it was highly reliable (Leech et al. 2015), similarly with Chinese (0.865), French (0.85) and Dutch (0.84) versions (Hudon et al. 2019; Lenferink et al. 2016; Xiaofei et al. 2017). In the original initial English study, the internal consistency value was found to achieve a good level (0.82) (Petkov et al. 2010). Studies show that similarities with the Turkish form were found. In a study conducted by Smith et al. (2017), the internal consistency value was 0.81 and 0.80 in the Spanish PIH validation study (de Peñarrieta‐ Córdova et al. 2014). With this result, the hypothesis ‘the reliability level of the Turkish form of the scale is high enough’ was confirmed.

When the correlations of the scale's total score and its sub‐dimensions were evaluated in the study, a very strong (if it is greater than 0.70) positive and statistically significant (*p* < 0.001) relationship was determined between the ‘knowledge’, ‘partnership in treatment’, ‘recognition and management of symptoms’ and ‘coping’ dimension and the overall scale. These relationships showed that the features of the measurement tool affected each other. According to Polit (2014), all of the sub‐dimensions showed a significant positive relationship to a moderate degree in the study (between 0.30 and 0.50) (*p* < 0.001). All sub‐dimensions have an overlapping structure since they include items reflecting the problems experienced by the patients, as well as their self‐management, knowledge and coping skills. The obtained relational values show that all dimensions complement one another and contribute to the scale. The study results are similar to the study by Smith et al. (2017) in that the correlations between the scale factors were moderately distributed at similar rates.

### 4.3 Limitations

In this research, sample groups were limited in that only outpatient level of care was included in the sample. Participants completed the questionnaire while sitting in the clinic waiting rooms. If the patient's appointment with the clinician was ahead of time and the participants did not have enough time to complete the questionnaire, it was rarely completed after that appointment.

## 5 Conclusion

The scale ‘Turkish version of the 12‐item Partners in Health Scale (PIH‐TR)’ was found to be reliable and exhibited satisfactory content and construct validity and strong test–retest reliability. Using the CFA model, it was determined that the four factor groups (knowledge, partnership in treatment, recognition and management of symptoms and coping) provided valid evidence with 12 items and goodness of fit indices, sufficiently aligned with findings from the analysis of the PIH Model‐2. The PIH‐TR can be used by health professionals to measure self‐management of CD in PHC Turkish‐speaking patients with chronic conditions. The PIH‐TR can improve health outcomes when used in standard care. It can evaluate service effectiveness and assess patients' self‐management needs.

Since the scale items were in a 9‐point Likert scale structure during the data collection phase and some patients were undecided in answering these items, the scale items could be analysed in a 7‐point Likert scale structure in future studies. It may be recommended to carry out validity and reliability tests by applying the scale to populations with different educational levels, age groups and specific to certain CDs in order to generalize it.

## Author Contributions

All authors contributed to the concept and design, acquisition and interpretation of data, drafting the article and gave final approval of the version to be published.

## Ethics Statement

Partners in Health Care Scale Licence Agreement was signed between author (JGY) and Flinders University research group (Lawn and other researchers). A university ethics committee permission was obtained (144‐2017), and written permission was obtained from the Provincial Health Directorate in Public Health (77597247‐604.02‐27.04.2018). The participants were informed verbally about the research procedures and that the answers to the questionnaire would be anonymous, and verbal consent was taken from the participants. The institutions were informed of the results obtained.

## Conflicts of Interest

The authors declare no conflicts of interests.

## Data Availability

The data that support the findings of this study are available on request from the corresponding author. The data are not publicly available due to privacy or ethical restrictions.

## Appendix A

**Table:** Turkish version of Partners in Health Scale (PIH‐TR)

Original version of revised PIH	Turkish translation of the revised PIH
1. Overall, what I know about my health condition(s). 2. Overall, what I know about the treatment, including medications of my health condition(s) is. 3. I take medications or carry out the treatments asked by my doctor or health worker. 4. I share in decisions made about my health condition(s) with my doctor or health worker. 5. I am able to deal with health professionals to get the services I need that fit with my culture, values and beliefs. 6. I attend appointments as asked by my doctor or health worker. 7. I keep track of my symptoms and early warning signs (e.g., blood sugar levels, peak flow, weight, shortness of breath, pain, sleep problems and mood) 8. I take action when my early warning signs and symptoms get worse. 9. I manage the effect of my health condition(s) on my physical activity (i.e., walking and household tasks) 10. I manage the effect of my health condition(s) on how I feel (i.e., my emotions and spiritual well‐being) 11. I manage the effect of my health condition(s) on my social life (i.e., how I mix with other people). 12. Overall, I manage to live a healthy life (e.g., no smoking, moderate alcohol, healthy food, regular physical activity and manage stress).	1. Genel olarak, hastalığımla ilgili bildiklerim 2. Genel olarak, hastalığımla ilgili ilaçlarım da dahil olmak üzere tedavim hakkında bildiklerim 3. Doktorumun ya da sağlık personelinin önerdiği tedavileri uygularım ve ilaçlarımı alırım 4. Sağlık durumumla ilgili kararları doktorum ya da sağlık personeli ile paylaşırım 5. Kültürüme, değerlerime ve inançlarıma uygun ihtiyacım olan sağlık hizmetlerini alma açısından sağlık personeli ile anlaşabiliyorum 6. Doktorumun ya da sağlık personelinin verdiği randevulara giderim 7. Hastalığımla ilgili erken belirti ve bulguları (Kan şekeri düzeyim, solunum fonksiyon testimin sonucu, kilom, nefes darlığı, ağrı, uyku sorunlarım, ruh halim gibi) takip ederim 8. Hastalığımla ilgili erken belirti ve bulgular kötüleştiğinde birşeyler yaparım 9. Hastalığımın fizik aktivitelerime getirdiği olumsuz etkilerle baş ederim. (Yürüyüş, ev işleri yapma gibi) 10. Hastalığımın hislerimi etkilemesini kontrol ederim. (Örneğin, duygularım, manevi iyilik halim gibi) 11. Hastalığımın sosyal hayatıma getirdiği olumsuz etkilerle baş ederim (örneğin diğer insanlarla kaynaşarak)12. Genel olarak, sağlıklı bir hayat sürdürebiliyorum (örneğin, sigara içmeyerek, ölçülü alkol tüketerek, sağlıklı beslenerek, düzenli fizik aktivite yaparak ve stresimi kontrol ederek)

PIH‐TR rated with a 9‐point Likert scale (0–8 points) the following descriptors of the scores: (a) *very poor*, to *a very good* in item 1–4, 6, 8, 10–12; (b) *never*, *sometimes*, to *always* in item 5, 7, 9. Higher scores indicate better self‐management. Twelve questions assessing four factors: Knowledge (items 1, 2), partnership in treatment (items 3–6), recognition and management of symptoms (items 7, 8), coping (items 9–12).
